# Supplementary material for: Diagnostics of BAP1-Tumor Predisposition Syndrome by a Multitesting Approach: A Ten-Year-Long Experience
Source: Diagnostics (Basel). 2022 Jul 13;12(7):1710. doi: 10.3390/diagnostics12071710 (PMC9317020; doi:10.3390/diagnostics12071710)
Supplement: Supplementary file 1 [file diagnostics-12-01710-s001.zip › diagnostics-1740979-supplementary.pdf]

SUPPLEMENTARY MATERIALS

Table S1. Clinical features of the patients (n=101) analyzed in the present study.

#	Tumor	If mesothelioma, histotype	Age at diagnosis	Family history
A_II-1 § (Family A)	PeM	Epithelioid	63	PlM (mother, sister), mucoepidermoid carcinoma (brother), meningioma (daughter)
1	PlM	Epithelioid	57	CM (brother), colon cancer (father)
2	PlM	Sarcomatoid	66	PlM (father, paternal cousin), lung cancer (paternal uncle)
3	PlM	Biphasic	49	PlM (mother)
4	PlM	Epithelioid	55	PlM (paternal grandmother), prostate cancer (father), unknown tumor (paternal aunt)
5	PlM	Epithelioid	70	PlM (mother, father)
6	PlM	Epithelioid	69	Lung cancer (mother)
7	PlM, Hodgkin's lymphoma	Epithelioid	28	Yes
8	PlM	Epithelioid	68	PlM (sister, niece/daughter of the sister, nephew /unknown)
9	PlM	Epithelioid	45	CM (brother), lung cancer (mother), primary gastric lymphoma (father), multiple myeloma (maternal grandmother)
10	PlM	Epithelioid	75	PlM (sister, paternal cousin, maternal cousin)
11	PlM, breast cancer	/	76	Yes
12	PlM, breast cancer	/	73	Yes
13	PlM	/	66	CM (daughter)
14	PlM	/	65	PlM (sister)
15	PlM, CM	Epithelioid	57	Yes
16	PlM	/	69	PlM (cousin/unknown)
ID_5 III-1 § (Family ID_%)	PlM	Epithelioid	52	Suspected PlM (mother)
A1_II-5 § (Family A1)	PlM, CM multiple, meningioma	Epithelioid	37	BCC (brother), CM (daughter), gastric cancer (mother), UM (brother)
17	PlM	Biphasic	53	PlM (two paternal cousins), PeM (paternal uncle), leukemia (paternal uncle)
18	CM, PlM, breast cancer	Epithelioid	51	Yes
19	PlM	Epithelioid	/	PlM (sister)
20	PlM	Epithelioid	72	PlM (brother), unknown tumor (brother)
21	PlM	Epithelioid	77	Breast cancer (sister)
22	PlM	Epithelioid	68	CM (daughter)
23	PlM	Epithelioid	76	PlM (paternal cousin)
24	Breast cancer, thyroid cancer, PlM	Epithelioid	39	Yes
25	PlM	Epithelioid	48	PlM (father), colon cancer (paternal uncle, paternal grandfather)
26	PlM, RCC, BCC	Epithelioid	73	Breast cancer (three sisters, nephew)
27	PlM	Epithelioid	52	Bladder cancer (father), breast cancer (maternal aunt), pancreatic cancer (maternal cousin)
MPM_HO1901 II-3 § (Family MPM_HO1901)	RCC, PlM, adenocarcinoma NOS	Epithelioid	46	PlM (brother), RCC (father), breast cancer (two paternal cousins), laryngeal cancer (mother), lung cancer (mother), subependymoma (brother)
28	PeM	Biphasic	70	Gastric cancer (brother, sister), breast cancer (sister), hepatocellular carcinoma (sister)
29	PlM, colon cancer	/	56	Gastric cancer (father), polyps (father)
30	PlM	/	67	Yes
31	PlM, breast cancer, lung cancer	Epithelioid	63	Yes
32	PlM	/	70	Laryngeal cancer (father, paternal uncle), breast cancer (paternal cousin), uterine cancer (cousin),

				lung cancer (father), pancreatic cancer (uncle/unknown)
33	PlM	Epithelioid	58	Lung cancer (father, two maternal uncles, maternal grandmother), sarcoma (nephew/son of the brother)
34	PlM	Epithelioid	83	PlM (sister)
35#	CM multiple	/	33	PlM (mother), breast cancer (maternal grandmother)
36#	CM, colon cancer, nasopharyngeal carcinoma	/	43	PlM (father)
37#	CM	/	42	CM (mother), M (mother)
38#	CM, breast cancer	/	30	M (father), breast cancer (mother), RCC (brother)
39#	UM (left, right), breast cancer	/	25	Acoustic neuroma (sister), bladder cancer (mother)
40#	UM	/	31	Yes
41#	CM, UM	/	67	CM (daughter, niece/daughter of the sister), lung cancer (father)
42#	UM, breast cancer	/	60	Breast cancer (sister, paternal aunt, maternal cousin), RCC (maternal aunt)
43#	CM	/	46	UM (father)
44#	CM multiple, RCC	/	43	Yes
45#	CM	/	65	CM (daughter, paternal cousin, nephew/son of the sister)
46#	CM multiple	/	54	CM (mother), RCC (sister), breast cancer (sister), cervical cancer (sister)
47#	CM multiple	/	35	RCC (father), CM (paternal uncle), lung cancer (two paternal uncles)
48#	CM multiple	/	28	non-Hodgkin's lymphoma (mother), CM (paternal uncle)
49#	CM multiple	/	43	CM (father)
50#	CM multiple	/	56	CM (father)
51#	CM multiple	/	67	CM (brother)
52#	CM	/	20	CM (mother)
53#	CM	/	24	CM (mother)
54#	CM	/	27	CM (father)
55#	CM	/	32	CM (sister)
56#	CM multiple	/	36	Thyroid cancer (father)
57#	CM multiple	/	39	Colon cancer (brother)
58#	CM	/	82	CM (brother), pancreatic cancer (sister)
59#	CM multiple, teratoma	/	39	Yes
60#	Adrenal adenoma, CM multiple	/	53	Prostate cancer (father), hepatocellular carcinoma (paternal uncle, paternal grandfather)
61#	CM	/	61	Breast cancer (sister, two paternal aunts, maternal cousin), lung cancer (paternal uncle)
62	PeM	Biphasic	49	PlM (two sisters)
63	PlM, neuroblastoma	Epithelioid	27	Yes
64	PlM, BCC	Epithelioid	79	Yes
65	Paraganglioma, seminoma	/	50	CM (twin sister), breast cancer (sister), colon cancer (mother), lung cancer (maternal cousin), leukemia (maternal cousin)
66	CM, breast cancer	/	27	CM (brother), breast cancer (two paternal aunts, paternal grandmother, paternal great grandmother, paternal great aunt), lung cancer (paternal grandfather, three paternal great uncles) prostate cancer (paternal great uncle), adenocarcinoma NOS (paternal great uncle), laryngeal cancer (paternal great uncle)
67#	CM multiple, RCC	/	32	Colon cancer (father, maternal uncle), gastric cancer (paternal grandmother)
68#	CM	/	40	CM (cousin, uncle), prostate cancer (cousin/unknown), pancreatic cancer (two paternal

				uncles), colon cancer (father, mother, paternal uncle), lung cancer (paternal maternal uncle), bladder cancer (uncle)
69#	CM multiple, breast cancer	/	47	Pancreatic cancer (brother, mother), colon cancer (mother)
70#	CM multiple, RCC	/	60	CM (daughter), breast cancer (mother, paternal aunt), lung cancer (father), thyroid cancer (father), prostate cancer (three paternal uncles), hepatocellular carcinoma (paternal uncle), Hodgkin’s lymphoma (paternal uncle), non-Hodgkin’s lymphoma (paternal uncle), esophageal cancer (paternal uncle)
71	Breast cancer, meningioma, RCC, colon cancer, thyroid cancer, BCC	/	59	Breast cancer (daughter, maternal aunt), brain cancer (maternal uncle)
72	RCC, myxoid chondrosarcoma	/	52	CM (brother), RCC (brother, maternal uncle, nephew)
73#	CM multiple, breast cancer	/	32	Yes
74	Breast cancer	/	68	PlM (brother, sister), prostate cancer (brother), breast cancer (sister), hepatocellular cancer (father), unknown tumor (mother, two maternal uncles)
75	UM	/	75	PlM (brother), CM (paternal cousin, two nephews/sons of the brothers), colon cancer (mother)
76#	CM	/	29	CM (maternal great grandfather), leukemia (maternal aunt)
77	BCC, CM, UM	/	74	Lung cancer (father), ovarian cancer (mother), lymphoma (mother), brain cancer (maternal grandfather), dysplastic nevi (two nephews/sons of the daughter)
78#	BCC, CM multiple, GIST	/	40	PlM (sister), pancreatic cancer (mother), unknown skin cancer (maternal grandmother)
79	CM	/	79	Brain cancer (mother), lung cancer (two sisters, maternal uncle, two maternal cousins, maternal aunt), pancreatic cancer (brother), endometrial cancer (maternal aunt), unknown tumor (two maternal cousins)
80#	CM, thyroid cancer, biliary tract cancer	/	58	CM (brother), uterine cancer (mother)
81#	CM	/	49	CM (brother), RCC (mother), bladder cancer (mother), breast cancer (mother), lung cancer (maternal grandfather), laryngeal cancer (maternal grandfather), gastric cancer (maternal grandmother), pituitary adenomas (cousin)
82	SM, breast cancer	/	48	Breast cancer (grandmother)
83	CM multiple	/	43	PlM (uncle), meningioma (mother), breast cancer (sister, paternal aunt, paternal grandmother),bladder cancer (father), lung cancer (maternal uncle, maternal grandfather), laryngeal cancer (maternal aunt)
84#	CM multiple	/	46	CM (brother), primary melanoma of the urinary bladder (father), prostate cancer (father)
85	Thyroid cancer	/	19	PlM (mother), gastric cancer (mother), BCC (paternal uncle), Hodgkin's lymphoma (paternal uncle), thyroid cancer (paternal uncle), prostate cancer (paternal grandfather), lung cancer (paternal grandfather), uterine cancer (paternal grandmother), leukemia (paternal great aunt), brain cancer (paternal great aunt), sarcoma (paternal great aunt)

86	Neuroendocrine tumor, BCC multiple, breast cancer, colon cancer	/	56	CM (brother), breast cancer (sister), lung cancer (father), laryngeal cancer (paternal uncle)
87#	CM	/	64	UM (father), pancreatic cancer (sister), gastric cancer (maternal cousin), unknown tumor (maternal cousin)
88	CM multiple	/	44	PlM (father), prostate cancer (paternal grandfather), uterine cancer (mother)
89	UM, adenocarcinoma	/	77	Acute leukemia (brother), breast cancer (mother), laryngeal cancer (maternal uncle)
90	CM	/	46	PlM (maternal aunt), breast cancer (mother, maternal grandmother), unknown peritoneal tumor (mother) brain cancer (maternal grandfather)
91	PlM, polyps	Epithelioid	67	PlM (sister), breast cancer (sister), hepatocellular carcinoma (sister)
92	Colon cancer	/	43	CM (father), adenoma (brother), breast cancer (mother, maternal cousin), gastric cancer (maternal grandfather, maternal great grandfather), prostate cancer (two paternal uncles, grandfather), Hodgkin's lymphoma (cousin), unknown tumor (paternal aunt)
93	UM, colon cancer	/	78	Yes
94	Ovarian cancer	/	36	PlM (father)
MM981# § (Family PD-578)	CM multiple, prostate cancer	/	79	PlM (niece/daughter of the sister), pancreatic cancer (sister), breast cancer (sister), prostate cancer (paternal uncle), unknown cancer (two maternal aunts)
MM1012# § (Family PD-601)	CM multiple	/	55	CM (maternal cousin), lung cancer (mother, paternal aunt), liver cancer (paternal grandmother), brain cancer (maternal uncle), leukemia (maternal aunt), womb cancer (maternal cousin), unknown cancer (paternal aunt, uncle)
MM400# § (Family PD-238)	UM, bladder cancer	/	58	UM (father, paternal aunt), brain cancer (paternal aunt), gastric melanoma (paternal uncle), lung cancer (two paternal uncles), colon cancer (paternal uncle).

§, carriers of a PVs in *BAP1*; #, wild type for *CDKN2A*, *CDK4*, *MITF*, *TERT*, *POT1*; M, mesothelioma; PeM, peritoneal mesothelioma; PlM, pleural mesothelioma; CM, cutaneous melanoma; UM, uveal melanoma; RCC, renal cell carcinoma; BCC, basal cell carcinoma; GIST, gastrointestinal stromal tumor; SM, spitzoid melanoma.

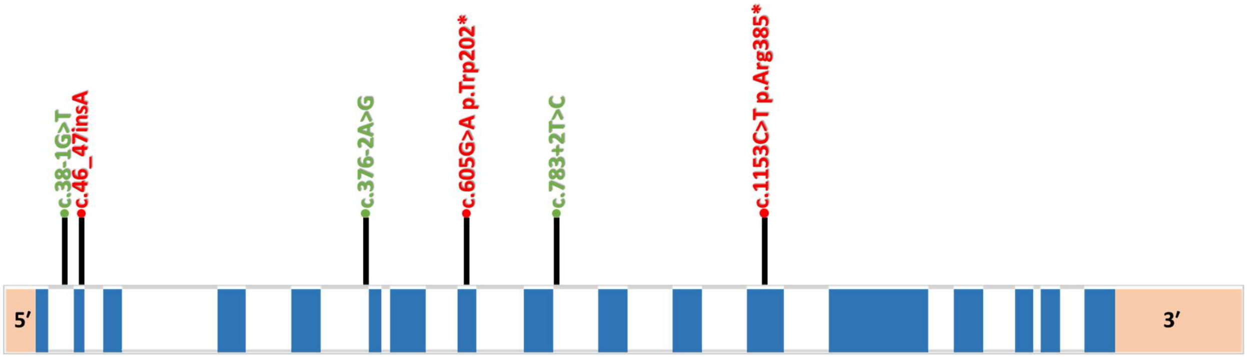

Figure S1. Lollipop plot of all variants discovered in this study mapped on the *BAP1* gene. White boxes represent introns, blue boxes represent exons. Splice site variants are in green, whereas stop gain/insertion variants are in red.

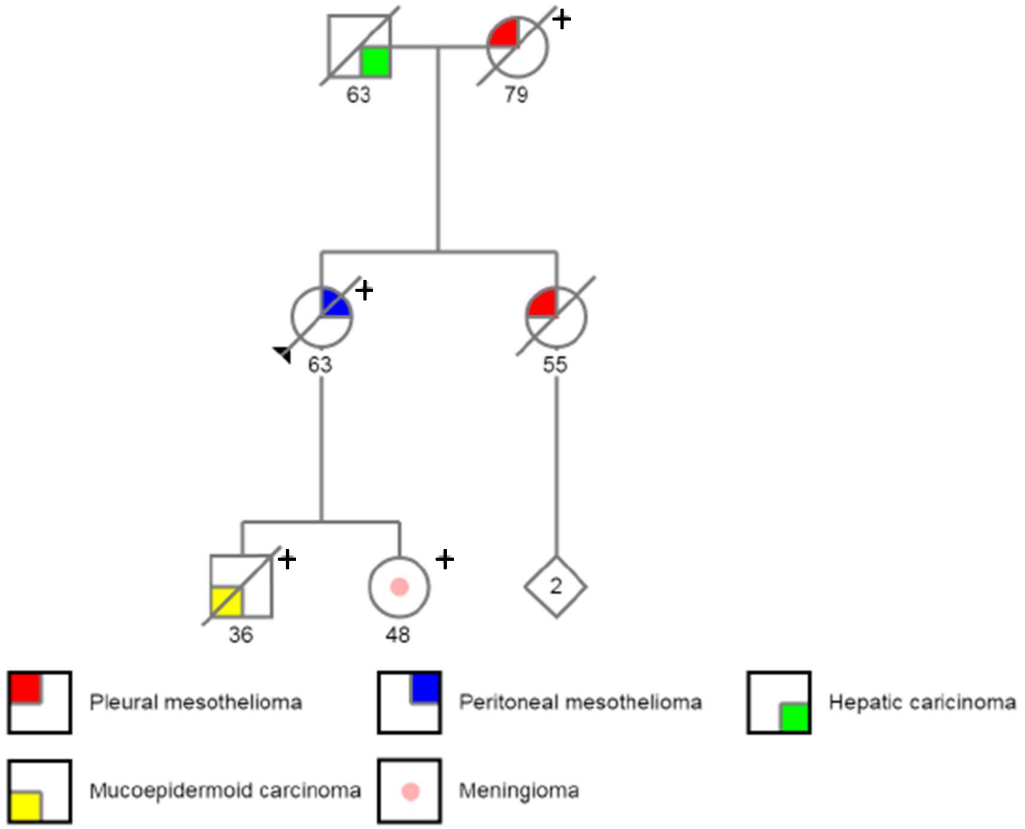

Figure S2. Pedigree of Family A. This is an update of the clinical features of Family A, previously reported by Betti *et al.* [1]. The black arrowhead indicates the proband. Carriers of the c.46_47insA *BAP1* variant are labeled with (+).

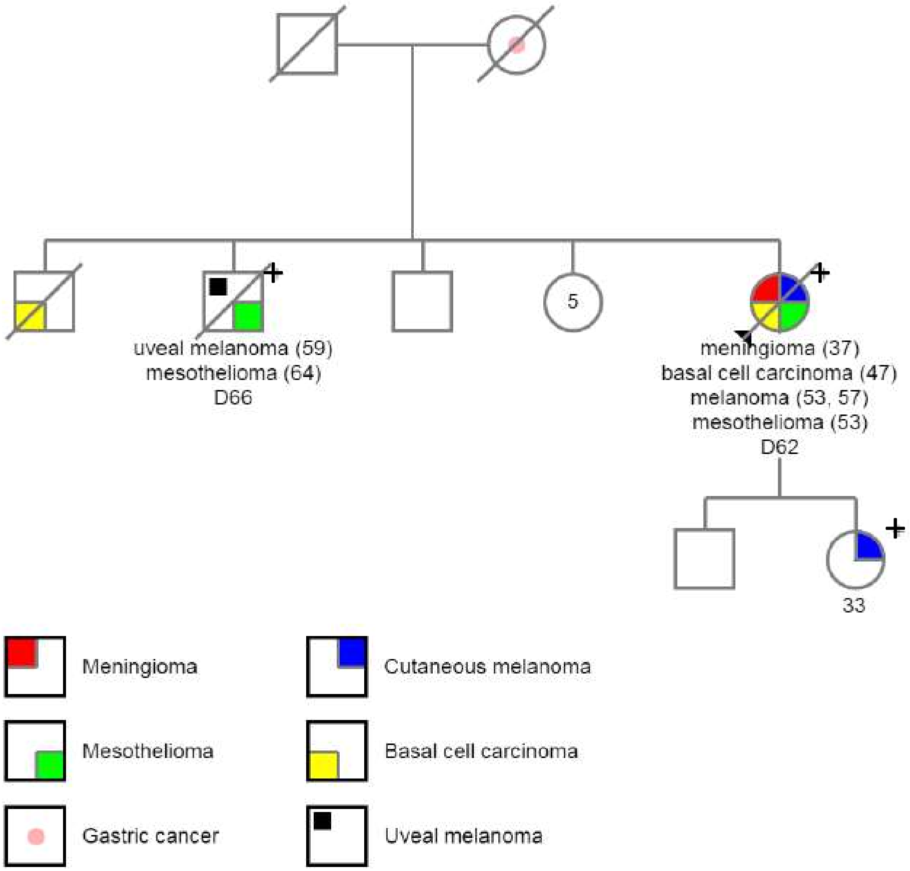

Figure S3. Pedigree of family A1. This is an update of the clinical features of family A1, previously reported by Betti *et al.* [2]. The black arrowhead indicates the proband. Carriers of the c.1153C>T p.Arg385* *BAP1* variant are labeled with (+).

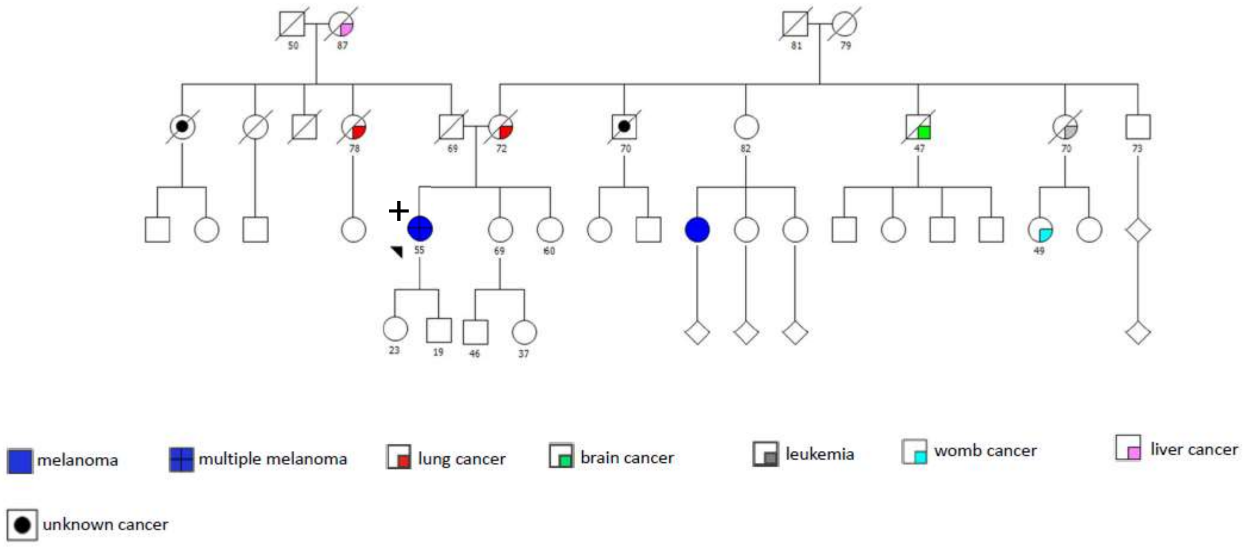

Figure S4. Pedigree of family PD-601. Proband MM1012 is indicated by the black arrowhead. She carries the c.783+2 G>T *BAP1* variant and is affected by multiple cutaneous melanomas (CMs). None of the other family members was tested by cascade analysis.

A

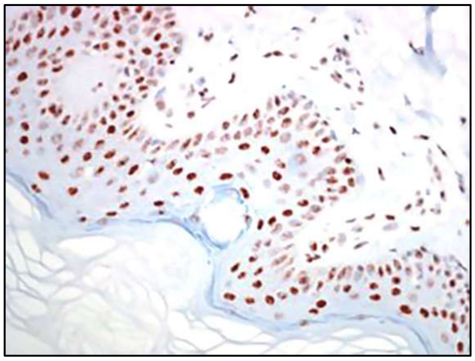

B-1

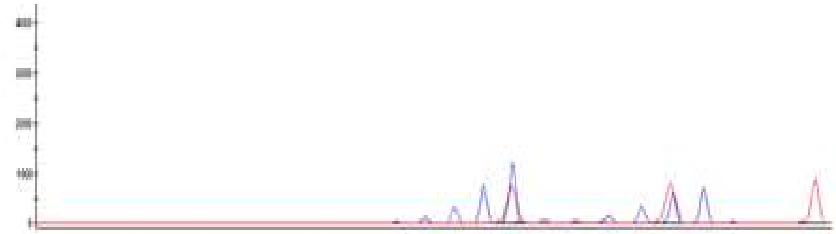

B-2

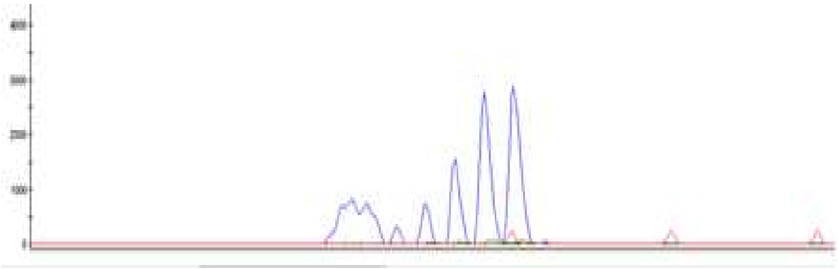

Figure S5. IHC and LOH analyses of tumor sample MM1012. (**A**) BAP1 is expressed in neoplastic atypical cutaneous and stromal cells. (**B**) Microsatellite analysis for detection of *BAP1* loss: **B-1**, germline DNA; **B-2**, tumor DNA.

A

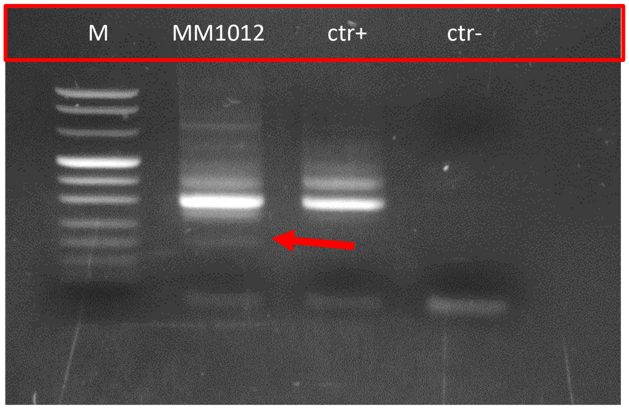

B

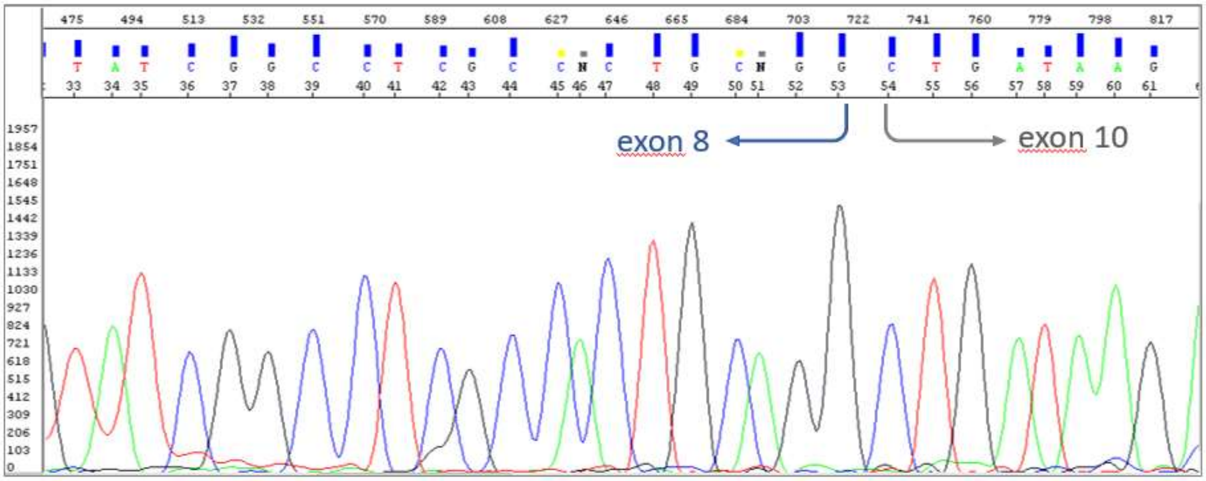

GGCCCTGGGGGGAGGACGAGGAGTGGACAGACAAGGCCCGGCGGGTCATCATGGAGCGTATCGGCCTCGCCACTGCAGG exon 8

GGAGCCCTACCACGACATCCGCTTCAACCTGATGGCAGTGGTGCCCGACCGCAGGATCAAGTATGAGGCCAGGCTGCATGT
GCTGAAGGTGAACCGTCAGACAGTACTAGAGGCTCTGCAGCAG exon 9

CTGATAAGAGTAACACAGCCAGAGCTGATTCAGACCCACAAGTCTCAAGAGTCACAGCTGCCTGAGGAGTCCAAGTCAGCC
AGCAACAAGTCCCCGCTGGTGCTGGAAGCAAACAGGGCCCCTGCAGCCTCTGAGGGCAACCACACAG exon 10

Figure S6. Functional analysis of the c.783+2 G>T variant from MM1012 blood samples. (**A)** Agarose gel showing the PCR products obtained from the cDNA of the MM1012 patient and of a healthy subject (ctr+). The red arrow indicates a faster migrating fragment that was purified and sequenced (**B**) revealing exon 9 skipping, that is predicted to cause frameshift and a premature stop codon in exon 10. Abbreviations: ctr+, control positive; ctr-, control negative; M, marker.

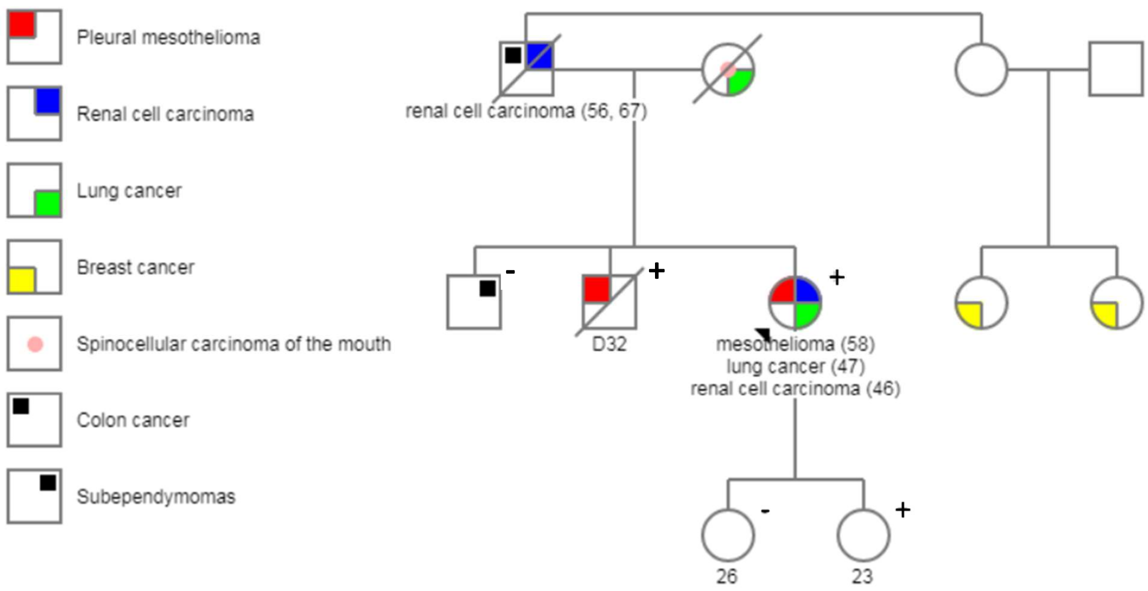

Figure S7. Pedigree of family MPM_HO1901. This is an update of the clinical features of family MPM_HO1901, previously reported by Sculco *et al.* [3]. The black arrowhead indicates the proband. Carriers of the c.38-1G>T *BAP1* variant are marked with (+). Family members negative for the variant are marked with (-).

A

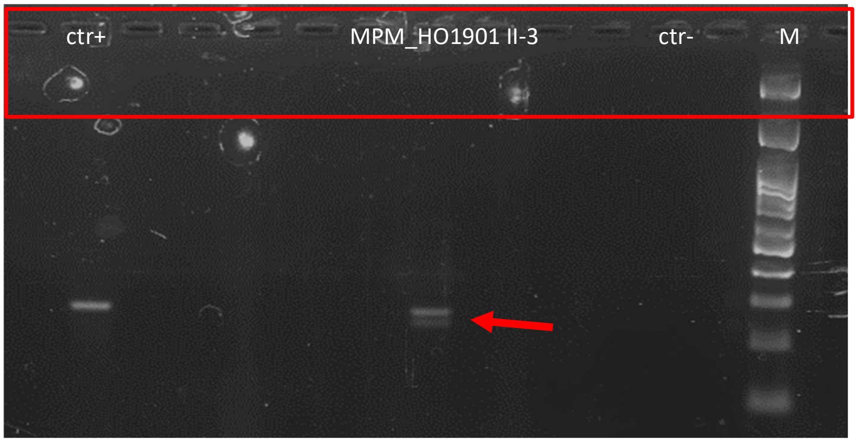

B

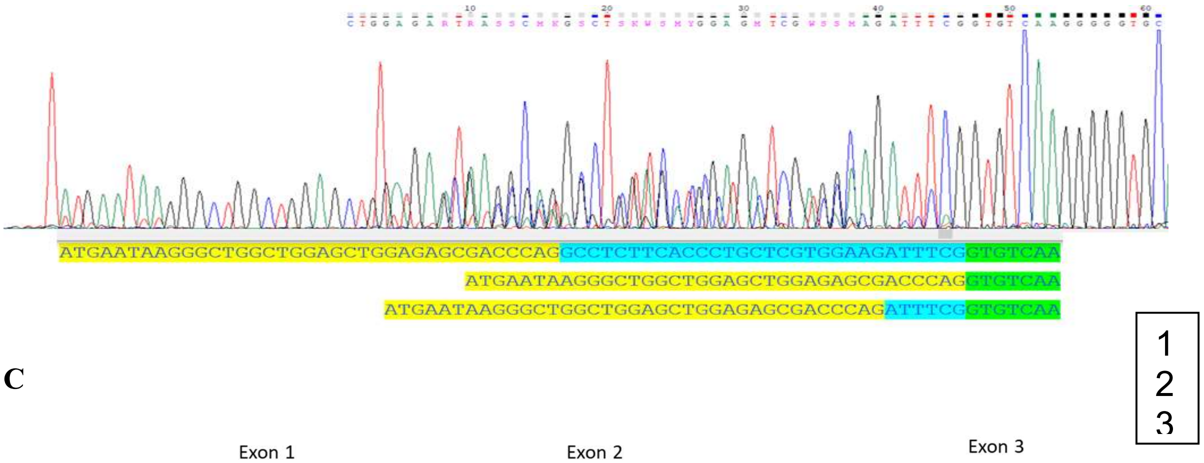

C

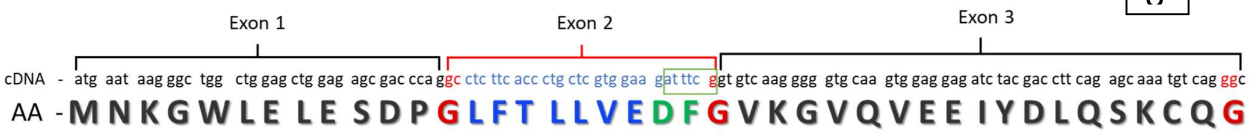

Figure S8. Functional analysis of the c.38-1G>T variant carried by MPM_HO1901. (**A**) Agarose gel showing the PCR products obtained from the cDNA of the MPM_HO1901 II-3 and a healthy subject (ctr+). The red arrow indicates a faster migrating fragment that was purified and sequenced using a reverse primer (**B**) revealing three different sequences: 1, wt; 2, exon 2 skipping; 3, cryptic splice site of exon 2. Exon 2 is highlighted in blue, exon 1 in yellow, while part of exon 3 is green. (**C**) Schematic graph of the products derived from the splice variants. Abbreviations: ctr+, control positive; ctr-, control negative, M, marker; AA, amino acids.

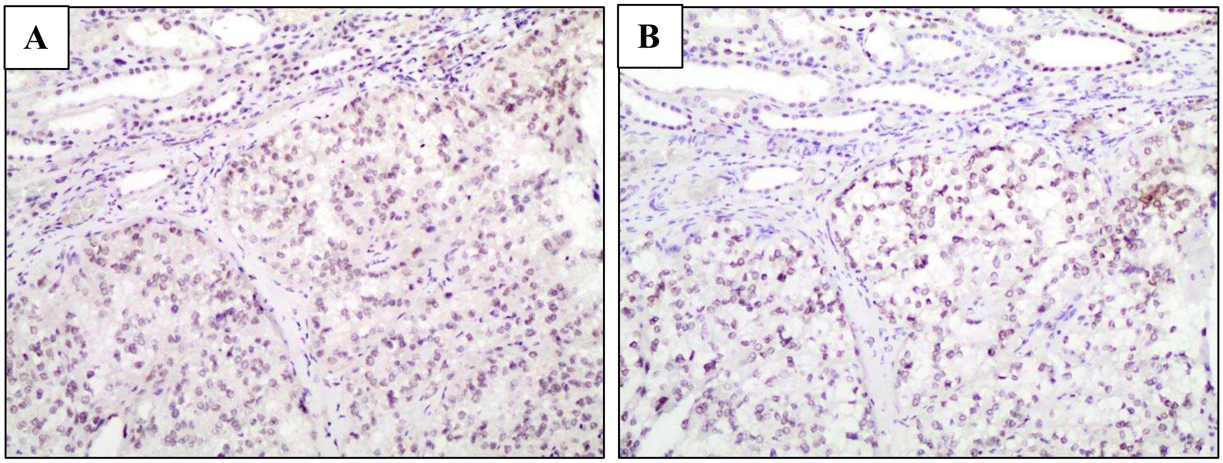

Figure S9. Histological and immunohistochemical features of proband MPM_HO1901 (II-3) RCC. RCC tumor cells showed heterogeneous BAP1 (**A**) and PAX8 (**B**) expression: both antigens showed the typical nuclear expression in tumor cells. Normal tubular cells served as internal control.

A

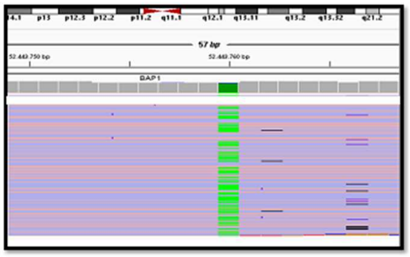

BAP1 germline variant, 89% of VAF in PlM

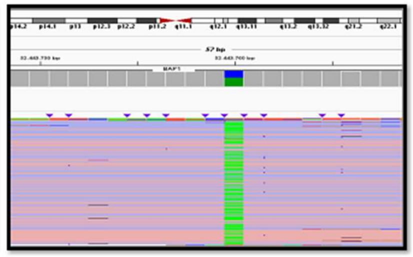

BAP1 germline variant, 48% of VAF in RCC

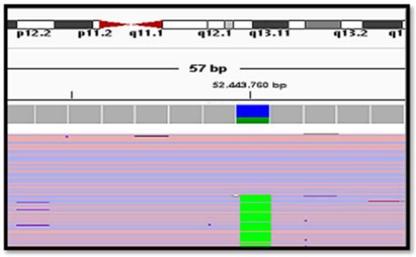

BAP1 germline variant, 21% of VAF in LUAD

B

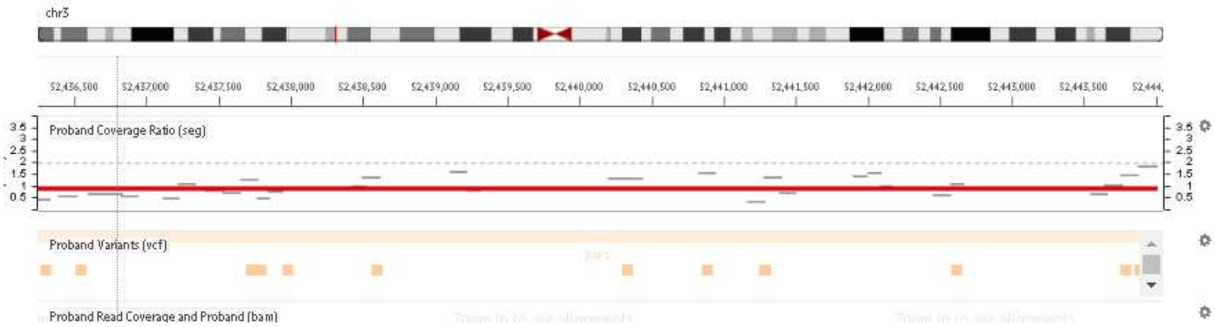

C

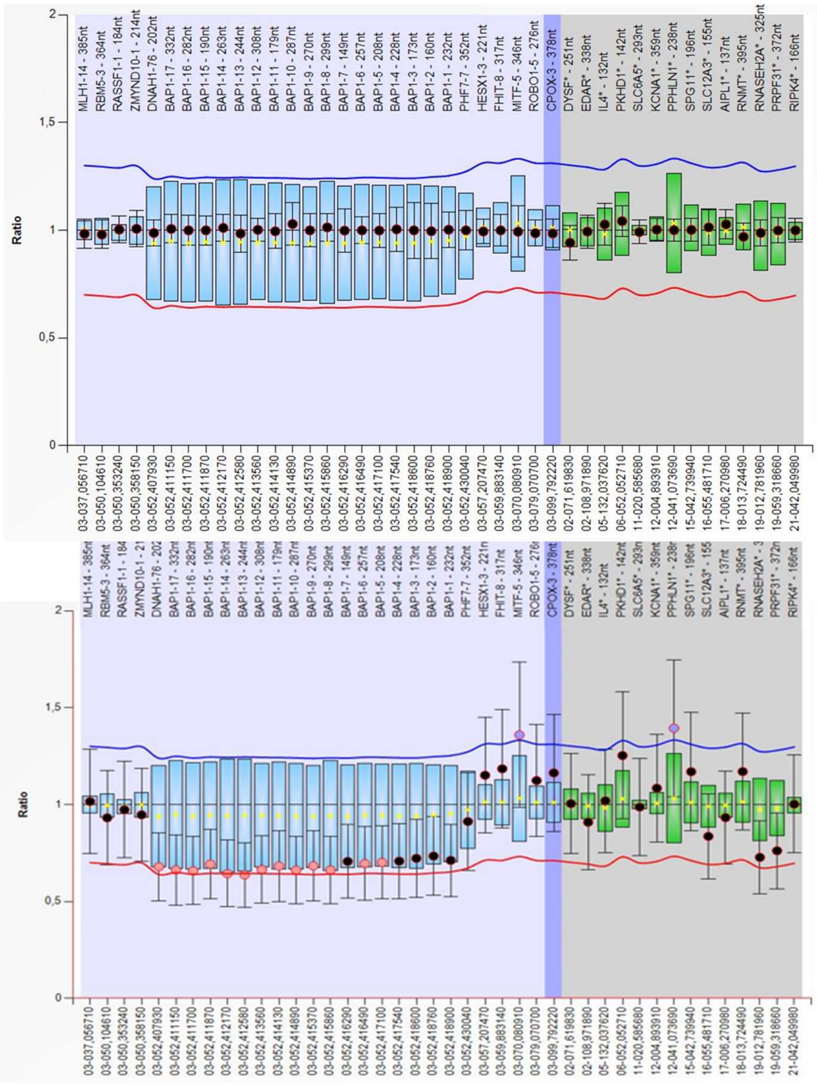

Figure S10. Molecular analyses of proband's (MPM_HO1901 II-3) PlM tissue sample. (**A**) NGS analysis and IRGV representation of *BAP1* (NM_004656.4) germline variation c.38–1G>A of the family MPM_HO1901. Proband's PlM, RCC, and LUAD FFPE tissue samples. (**B**). Schematic representation of CNV detected by NGS analysis. The red bar represents the mean of the ploidy value (0.96) found in the chromosome 3 call (52436209-52444009) from the proband's PlM tissue sample (normal ploidy value set at 2), thus suggesting a complete allele loss. (**C**) MLPA results comparing germline DNA controls (upper box) with DNA extracted from pleural effusion cell cultures from patient MPM_HO1901 II-3 (lower box). The ratio chart shows the heterozygous deletion of the whole *BAP1* gene.

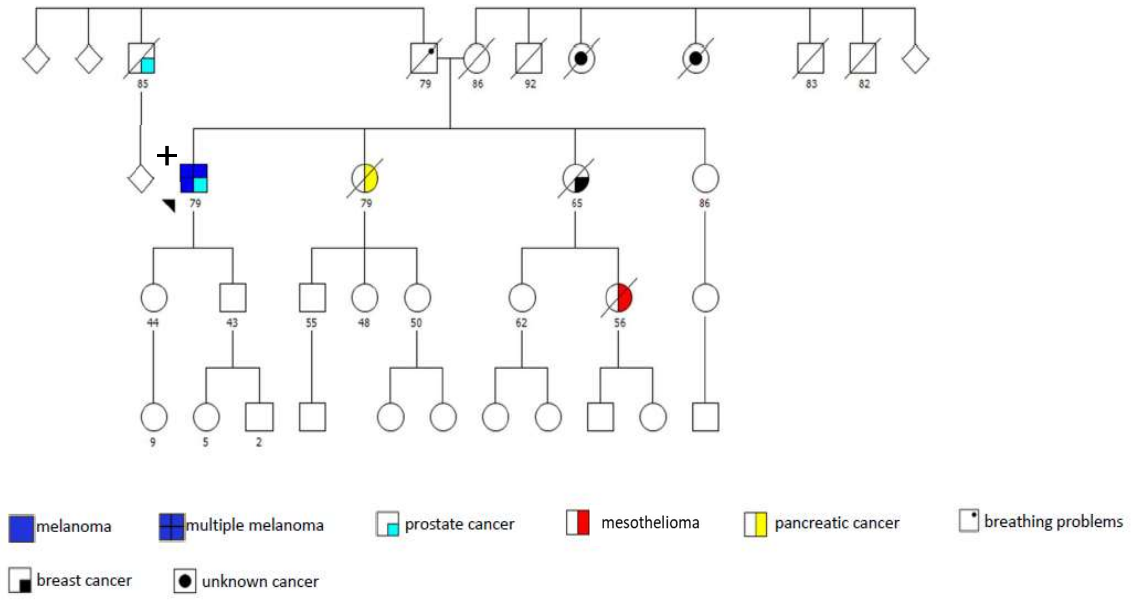

Figure S11. Pedigree of family PD-578. Proband MM981 is indicated by the black arrowhead. He carries the c.605G>A p.Trp202* *BAP1* variant and is affected by multiple CMs and prostate cancer. None of his family member was tested by cascade analysis.

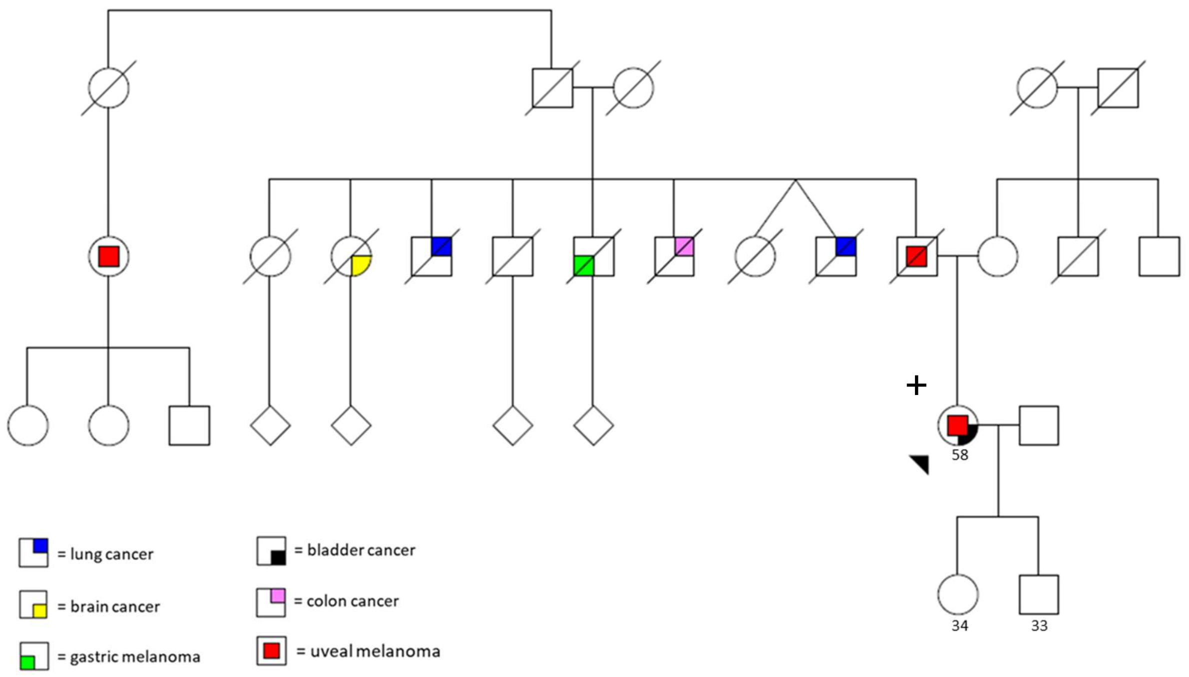

Figure S12. Pedigree of family PD-238. Proband MM400 is indicated by the black arrowhead. She carries the c.376-2A>G *BAP1* variant and is affected by UM and bladder cancer. None of the other family member was tested by cascade analysis. The variant was previously reported in an Australian family [4].

A

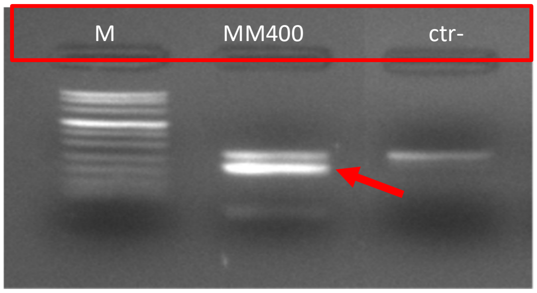

B

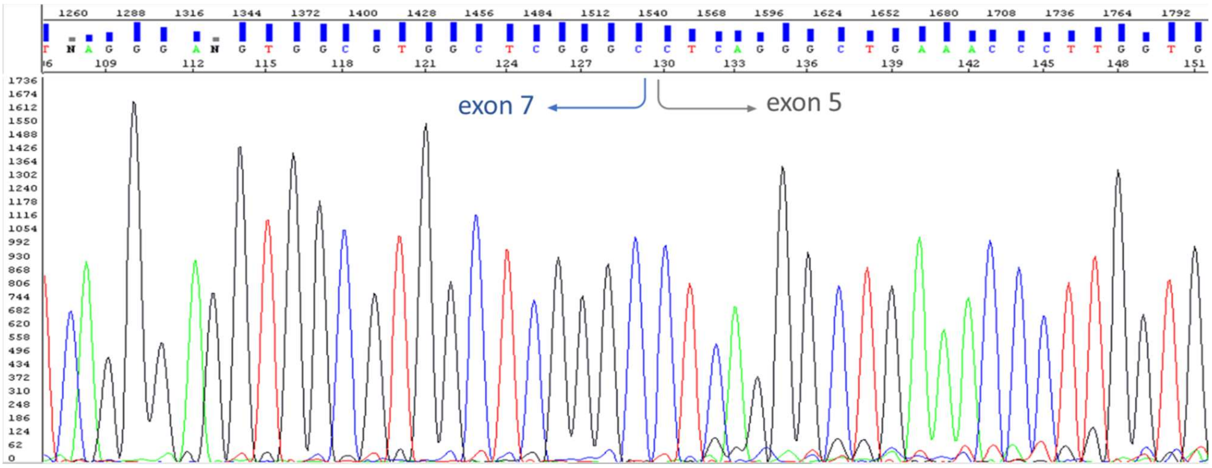

CATGGTCAATGGGGTAGACCTTCAGCCCATCCAGCTCAAAGAGCCGGCCTGTGATAGGCACATAGCTGACAAA
GTGGAACGCCTCCATGGTCCGCACTGCACTAAGGCCATTCTGCTTCTCAGGGAGGTGGCGTGGCTCGGGC exon 7

CTGGCATGGCTATTATGGGCCTTGGCCAACTCCGGGGCATTGCCAATCGCATATCCTTTGCT exon 6

CTCAGGGCTGAAACCCTTGGTGAAGTCCTTCATGCGACTCAGGGTGGGTCCCAGGTCCACGCTGCTGCAGTT
CAGGAGCACGCTCAGCAAGGCATGAGTTGCACAAGAGTTGGGTATCAG exon 5

Figure S13. Functional analysis of the variant carried by MM400. **A**. Agarose gel showing the RT-PCR products obtained from cDNA of the MM400 transcript and of a healthy subject (ctr+). The red arrow indicates the altered fragment that was purified and sequenced (**B)** showing exon 6 skipping. Abbreviations: ctr+, control positive, M, marker.

Supplemental Data

MSH6 Sanger sequencing, IHC, and MSS analyses

The p.Arg1076His *MSH6* variant identified in the tumor samples from HO19.01 (II-3) and her brother (II-2) by NGS was validated by Sanger sequencing on the germline DNAs of the two subjects. The following custom primers were used for amplification and sequencing:

F 5'- CCAAACGATGAAGCCTCAC -3'

R 5'- TGCAAGGATGGCGTGATCC -3'.

IHC analysis was performed in all tumor samples of MPM_HO19.01 (II-3) as follows: 5 mm-thick serial paraffin sections from representative paraffin blocks were processed using an automated platform (OMNIS, Dako) with CE-IVD Ready-to-Use primary antibody hMSH6 (Clone EP49, cod.IR08661-2). From five-μm-thick sections, DNA was extracted through an automated system (Maxwell RSC Instrument, Promega, Italy) using a specific Maxwell RSC DNA FFPE Kit (Promega, Italy), and it was subsequently evaluated by QuantusFluorometer (Promega Italia S.r.L., Milano).

To assess the MSI status, an Easy-PGX ready MSI (Diatech Pharmacogenetics, Jesi, Italy) kit was used. This real-time PCR-based assay allows the analysis of 8 mononucleotide repeats microsatellite markers (BAT-25, BAT-26, NR-21, NR-22, NR-24, NR-27, CAT-25 and MONO-27) in eight different reactions. Each real-time PCR reaction was performed in 25 μL, according to the manufacturer's instructions, using 30 ng of DNA. PCR products were analyzed by Agilent Aria Software v1.4 and evaluated by Easy-PGX qPCR Instrument 96 and EasyPGX Analysis Software v.3.0.0 (Diatech Pharmacogenetics, Jesi, Italy). According to the revised Bethesda guidelines, samples were considered to be microsatellite stable (MSS) if all microsatellites presented no length changes. In contrast, samples in which one or ≥ 2 microsatellites were altered were given, respectively, a low microsatellite instability (MSI-L) or a high microsatellite instability (MSI-H) status [5].

References

1. Betti, M.; Casalone, E.; Ferrante, D.; Romanelli, A.; Grosso, F.; Guarrera, S.; Righi, L.; Vatrano, S.; Pelosi, G.; Libener, R.; et al. Inference on Germline BAP1 Mutations and Asbestos Exposure from the Analysis of Familial and Sporadic Mesothelioma in a High-Risk Area. *Genes. Chromosomes Cancer* **2015**, *54*, 51–62, doi:10.1002/gcc.22218.
2. Betti, M.; Aspesi, A.; Biasi, A.; Casalone, E.; Ferrante, D.; Ogliara, P.; Gironi, L.C.; Giorgione, R.; Farinelli, P.; Grosso, F.; et al. CDKN2A and BAP1 Germline Mutations Predispose to Melanoma and Mesothelioma. *Cancer Lett.* **2016**, *378*, doi:10.1016/j.canlet.2016.05.011.
3. Sculco, M.; La Vecchia, M.; Aspesi, A.; Pinton, G.; Clavenna, M.G.; Casalone, E.; Allione, A.; Grosso, F.; Libener, R.; Muzio, A.; et al. Malignant Pleural Mesothelioma: Germline Variants in DNA Repair Genes May Steer Tailored Treatment. *Eur. J. Cancer* **2022**, *163*, 44–54, doi:10.1016/J.EJCA.2021.12.023.
4. Walpole, S.; Pritchard, A.L.; Cebulla, C.M.; Pilarski, R.; Stautberg, M.; Davidorf, F.H.; de la Fouchardière, A.; Cabaret, O.; Golmard, L.; Stoppa-Lyonnet, D.; et al. Comprehensive Study of the Clinical Phenotype of Germline BAP1 Variant-Carrying Families Worldwide. *J. Natl. Cancer Inst.* **2018**, doi:10.1093/jnci/djy171.
5. Umar, A.; Boland, C.R.; Terdiman, J.P.; Syngal, S.; de la Chapelle, A.; Rüschoff, J.; Fishel, R.; Lindor, N.M.; Burgart, L.J.; Hamelin, R.; et al. Revised Bethesda Guidelines for Hereditary Nonpolyposis Colorectal Cancer (Lynch Syndrome) and Microsatellite Instability. *J. Natl. Cancer Inst.* **2004**, *96*, 261–268, doi:10.1093/JNCI/DJH034.
